# Alpha-Glucosidase Inhibitory Activity of Saponins Isolated from *Vernonia gratiosa* Hance

**DOI:** 10.4014/jmb.2212.12040

**Published:** 2023-03-06

**Authors:** Pham Van Cong, Hoang Le Tuan Anh, Le Ba Vinh, Yoo Kyong Han, Nguyen Quang Trung, Bui Quang Minh, Ngo Viet Duc, Tran Minh Ngoc, Nguyen Thi Thu Hien, Hoang Duc Manh, Le Thi Lien, Ki Yong Lee

**Affiliations:** 1 Graduate University of Science and Technology, VAST, Hanoi 122000, Vietnam; 2 Center for Research and Technology Transfer (CRTT), Vietnam Academy of Science and Technology (VAST), 18 Hoang Quoc Viet, Hanoi 100000, Vietnam; 3 College of Pharmacy, Korea University, Sejong 30019, Republic of Korea; 4 Institute of Marine Biochemistry, VAST, Hanoi 122000, Vietnam; 5 National Institute of Medicinal Materials (NIMM), 3B Quang Trung, Hoan Kiem, Hanoi 12100, Vietnam; 6 Traditional Medicine Administration Ministry of Health, 138 Giang Vo, Ba Dinh, Hanoi 100000 Vietnam; 7 Hanoi University of Mining and Geology, Pho Vien, Duc Thang, Bac Tu Liem, Hanoi 129000, Vietnam; 8 Mientrung Institute for Scientific Research, VAST, Huynh Thuc Khang, Thua Thien Hue 52000, Vietnam

**Keywords:** *Vernonia gratiosa*, stigmastane-type steroidal glycoside, α-glucosidase inhibitor, vernogratiosides A–C

## Abstract

Species belonging to the *Vernonia* (Asteraceae), the largest genus in the tribe Vernonieae (consisting of about 1,000 species), are widely used in food and medicine. These plants are rich sources of bioactive sesquiterpene lactones and steroid saponins, likely including many as yet undiscovered chemical components. A phytochemical investigation resulted in the separation of three new stigmastane-type steroidal saponins (1 – 3), designated as vernogratiosides A–C, from whole plants of *V. gratiosa*. Their structures were elucidated based on infrared spectroscopy (IR), one-dimensional (1D) and two-dimensional nuclear magnetic resonance (2D NMR), high-resolution electrospray ionization mass spectrometry (HR-ESI-MS), and electronic circular dichroism analyses (ECD), as well as chemical reactivity. Molecular docking analysis of representative saponins with α-glucosidase inhibitory activity was performed. Additionally, the intended substances were tested for their ability to inhibit α-glucosidase activity in a laboratory setting. The results suggested that stigmastane-type steroidal saponins from *V. gratiosa* are promising candidate antidiabetic agents.

## Introduction

Diabetes occurs due to insulin resistance or reduced production of insulin by the pancreas [1]. The World Health Organization (WHO) reports that in 2016, diabetes was responsible for 1.6 million deaths globally. Diabetes is expected to become the sixth leading cause of death by 2030 [2]. Hyperglycemia often involves diabetes mellitus (DM), which is related to a number of diseases including atherosclerosis, cardiovascular disease, gastrointestinal disorders, and stroke [3].

Symptoms of type 2 diabetes include hypertension, blindness, renal failure, and heart disease, all of which result from elevated blood sugar levels [4]. α-Glucosidase (E.C. 3.2.1.20), which is located on the epithelium of the small intestine, is an enzyme that breaks down the terminal glucose glycosidic connections in disaccharides and polysaccharides [5]. α-Glucosidase plays a vital role in raising the blood sugar level and is therefore a target for the development of new treatments for type 2 diabetes [6]. Increased glucose levels after a meal are linked to the catalytic effect of the enzyme alpha-glucosidase on carbohydrates. Consequently, targeting this enzyme is an efficient method for managing and treating DM. Natural substances remain a significant factor in the process of developing drugs, with bioactive chemicals discovered from historically medicinal herbs serving as major sources of therapeutic agents for a number of diseases. The development of α-glucosidase inhibitors from natural products, such as acarbose, miglitol, and nojirimycin, has been crucial for managing type 2 diabetes [7].

Historically, natural substances found in products have been the most important source for the discovery of new potential pharmaceuticals [8]. Up to now, molecular modeling (*in silico*) has been the most widely applied tool for structure-based drug exploration. Indeed, rapidly determining potential target protein inhibitors, low cost, and great flexibility were the advantages of this method [9, 10]. Maltase-glucoamylase (MGAM) has been identified as the enzyme responsible for the final glucose-releasing step of starch digestion [11]. From a nutritional perspective, research on MGAM is significant because little is known about the intestinal processing of amylase-digested dietary components. Previously, triterpenoids isolated from the leaves of *Actinidia arguta* were reported an antidiabetic property via analysis of molecular docking results from two types of human maltase-glucoamylase (NtMGAM and CtMGAM) [12].

The genus *Vernonia* (Asteraceae) includes more than 1,000 species with a worldwide distribution, which are used as food and traditional medicine, and for industrial applications [13]. The stigmastane-type steroids have a molecular structure composed of 17 carbon atoms organized in four rings, and 10 carbon atoms in their side chains; they are found primarily in *Vernonia* species, and are divided into classes Δ^7,9(11)^ and Δ^8,9(14)^ based on the location of the double bond within the molecule. Several Δ^7,9(11)^ stigmastane-type steroid saponins were reported as major components in *Vernonia* species, including vernoniosides (A1–A3) and (B1–B3) [14]. These stigmastane-type steroids from *Vernonia* species have numerous pharmacological properties, including antiinflammatory, antidiabetic, antitumor, and antimalarial effects [15]. Up to now, phytochemical and biological studies have focused on *V. amygdalina*, *V. anthelmintica*, and *V. cinerea* [15]. However, there have been few studies of the chemical components of *V. gratiosa*. Our previous study demonstrated that compounds isolated from *V. gratiosa* have potential inhibitory activity, targeting the main protease of severe acute respiratory syndrome coronavirus 2 [16]. Here, we report the extraction, purification, and structural determination of three new Δ^7,9(11)^ stigmastane-type steroid saponins (**1–3**) from whole *V. gratiosa* plants. Furthermore, molecular docking analysis and in vitro experiments were performed to determine their α-glucosidase inhibitory activity.

## Materials and Methods

### Instrumentation and Reagents

The circular birefringence was measured on a P-2000 Digital Polarimeters. HR-ESI-MS spectrum was measured on an X500 QTOF mass spectrometer (Sciex, USA). IR spectra were measured on an Agilent 6530 Accurate-Mass spectrometer. The 1D and 2D NMR including ^1^H-(500 MHz), and ^13^C-(125 MHz) NMR spectra were employed using AVANCE III HD 500 FT-NMR spectrometer (Bruker, Corp., Germany). CD spectrum was achieved using Applied Photophysics Chirascan spectropolarimeter. Column chromatography (CC) was performed using silica gel (60F245, and RP-18 F254s. Merck, Germany), Sephadex LH-20 (Sigma-Aldrich, USA), and Thin Layer Chromatography (TLC) was performed on silica gel 60 F254 [23]. The identified compounds were monitored under a UV lamp at 254 or 365 nm and heated immediately after spraying with 10 % H_2_SO_4_.

### Plant Material

The raw materials of *V. gratiosa* used in this study were harvested in Huong Hoa, Quang Tri region, Vietnam, during April 2019 and verified by Dr. Anh TTP, VAST, Vietnam. The reference sample was kept at the Herbarium of CRTT, VAST under the code VG-2020.

### Purification of Compounds

Whole dried *V. gratiosa* plants (5.0 kg) were subjected to ultrasonic extraction with MeOH (5L each time) three times (2.5 h each time) at ambient temperature. The extract was concentrated under low pressure to give MeOH residue (400.0 g), which was further suspended in H_2_O (1.0 L) and successively partitioned with *n*-hexane (3 × 2.0 L), dichloromethane (CH_2_Cl_2_, 3 × 2.0 L), and ethyl acetate (EtOAc, 3 × 2.0 L) to give *n*-hexane (70.0 g), CH_2_Cl_2_ (40.0 g), EtOAc (42.0 g), and water residue (200.0 g), respectively. The water (W, 200.0 g) was fractionated over Diaon HP-20 with MeOH-H_2_O ratios of 25:75, 50:50, 75:25, and 100:0, (v/v) to afford four fractions (W1–W4). Subfraction W4 (31.0 g) was applied to a silica gel column with a gradient of CH_2_Cl_2_/MeOH (20/1 to 1/1, v/v) to afford five fractions (W4A-W4E) based on TLC guidance.

Subfraction W4A (9.0 g) was isolated by RP-18 with MeOH/H_2_O (2/1, v/v) to get compound **3** (11.0 mg). Subfraction W4C (1.8 g) was isolated by RP-18 chromatography with MeOH/H_2_O (2/1, v/v) as the solvent, followed by passage over a Sephadex LH-20 column using MeOH as the solvent to afford compounds **1** (9.0 mg) and **2** (60 mg).

### Vernogratioside A (1)

White amorphous powder; [α]_D_^25^ -28 (*c* 0.1, MeOH); IR (KBr) *ν_max_* cm^-1^: 3406, 2940, 2876, 1708, 1687, 1420, 1053; CD (*c* 4.5 × 10^-4^, MeOH) λ_max_ (mdeg) 221 (+2.82), and 243 (+10.78) nm; The detailed NMR data were shown at Table 1. HR-ESI-MS: *m/z* [M+Cl]- 831.3892 (calcd for C_41_H_64_ClO_15_^-^ 831.3939).

### Vernogratioside B (2)

White amorphous powder; [α]_D_^25^ -32 (*c* 0.1, MeOH); IR (KBr) *ν_max_* cm^-1^: 3406, 2936, 2878, 1706, 1688, 1437, 1052; CD (*c* 5×10^-4^, MeOH) λ_max_ (mdeg) 220 (+3.74), and 243 (+8.10) nm; The detailed NMR data were shown at Table 1. HR-ESI-MS: *m/z* [M-H]^−^ 779.4212 (calcd for C_41_H_63_O_14_^−^ 779.4223); and *m/z* [M+Cl]^−^ 815.3984 (calcd for C_41_H_64_ClO_14_^−^ 815.3990).

### Vernogratioside C (3)

White amorphous powder; [α]_D_^25^ -33 (*c* 0.1, MeOH); IR (KBr) *ν_max_* cm^-1^: 3408, 2930, 2877, 1705, 1686, 1457, 1054; CD (*c* 5×10^-4^, MeOH) λ_max_ (mdeg) 221 (+3.17), and 243 (+9.18) nm; The detailed NMR data were shown at Table 1. HR-ESI-MS: *m/z* [M+H]^+^ 797.4316 (calcd for C_41_H_65_O_15_^+^ 797.4318); and [M+Na]^+^ 819.4140 (calcd for C_41_H_64_O_15_Na^+^ 819.4137).

### Acid Hydrolysis and Sugar Identification

Acid hydrolysis and absolute sugar identification were performed as described previously [17, 24]. Briefly, aliquots of compounds **1–3** (3.0 mg) were refluxed with 1–5 ml of 1 N HCl for eight hours. Upon cooling, the solvent was removed by evaporation under a flow of nitrogen gas, and the remaining substance was divided through a CH_2_Cl_2_ and water solvent-solvent partitioning method. The single sugar found in the results of hydrolysis was obtained by preparative TLC using mobile phase as dichloromethane-methanol-water ratios of 2:1:0.2, and the circular birefringence was measured immediately. The circular birefringence values (${\left[\text{α}\right]}_{\text{D}}^{\text{20}}$) of monosaccharide were identified and compared to the reference. The existence of D-glucose and D-galactose in the acid hydrolysis products of metabolites **1–3** was verified by TLC and contrast of their circular birefringence to authentic standards, D-glucose [Rf 0.30,${\left[\text{α}\right]}_{\text{D}}^{\text{20}}$ = +20.6 (*c* 0.1, H_2_O)] and [Rf 0.35, ${\left[\text{α}\right]}_{\text{D}}^{\text{20}}$= + 46.5 (*c* 0.1, H_2_O)], as reported previously [25, 26].

### Molecular Docking Analysis

Molecular docking simulation was carried out by SYBYL-X 2.1.1 software (Tripos Ltd., USA) with two representative α-glucosidase proteins, *i.e.*, the crystal structures of human MGAM *N*-terminal subunit (NtMGAM; PDB-ID: 2QMJ) and C-terminal subunit (CtMGAM; PDB-ID: 3TOP) [21]. To create the protein, water was eliminated, the original ligands were removed, missing residues were repaired, and polar hydrogen atoms were added by the Tripos force field. In SYBYL-X 2.1.1, the ligand was prepared using the “Sanitize” process. The binding affinity was measured based on the total score, with higher scores indicating stronger protein–ligand binding affinity. The position of the ligand in the docked complex was visualized using BIOVIA Discovery Studio 2020 software (Dassault Systèmes, USA).

### α-Glucosidase Inhibitory Assay

A technique reported previously was used to identify α-glucosidase inhibitory activity [5]. Briefly, a reaction mixture (total volume = 60 μl) containing 100 M phosphate buffer (pH 6.8, 20 μl), p-NPG (2.5 mM, 20 μl), and the test compounds in 10% DMSO was added to the wells of 96-well plates. Then, 20 μl of α-glucosidase buffer was added to each well, mixed, and incubated at 37°C for 15 min before adding sodium carbonate solution to terminate the reaction. The activity of α-glucosidase was assessed by measuring the absorption at wavelength of 405 nm on a UV-Vis spectrophotometer. Acarbose was employed as a reference for comparison [27]. The inhibition of α-glucosidase activity was calculated using the equation below:

Inhibitory activity (%) = [(ΔC-ΔI)/ΔC] × 100; Where C and I were the intensity of control and inhibitors, respectively.

### Statistical Analyses

Data were identified using two-way ANOVA followed by Dunnett’s multiple comparison test and groups were thought to be significant if *p* < 0.05. All data are presented as means ± SD.

### Date Availability

The NMR, and HR-ESI-MS spectra of isolated compounds can be found in this article. Other information about this research is available upon request from the corresponding author.

## Results and Discussion

### Strutural Elucidation of Compounds 1–3

**Fig. 1:**
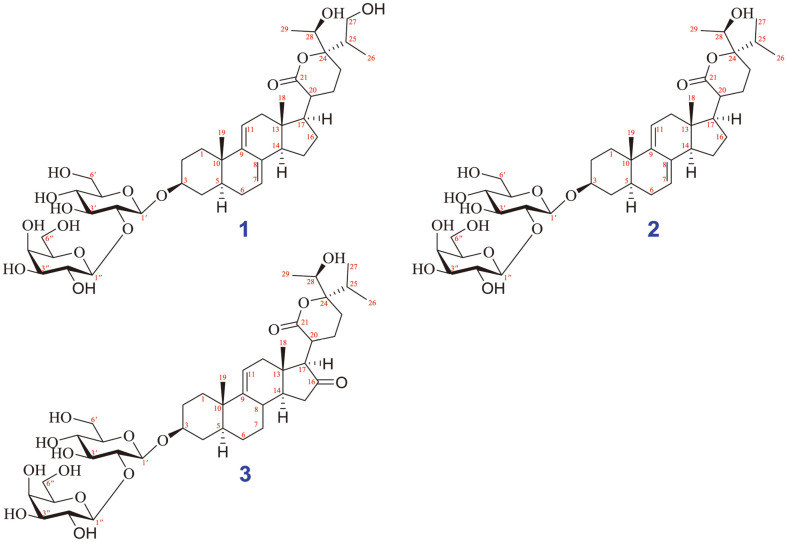
Structure of isolated compounds (1-3) from *V. gratiosa*. Compounds **1-3** were vernogratiosides A-C, respectively.

The methanolic crude extract was divided into different fractions based on their polarity through a solvent-solvent partition using polar (water), semipolar (ethyl acetate), and non-polar (hexane) solvents. This was done in order to isolate various classes of active components. The water layer faction contains saponin components [17, 18]. Thus, the methanol (MeOH) extract of whole plants of *V. gratiosa* was partitioned with solvents with increasing levels of polarity, including *n*-hexane, dichloromethane, and ethyl acetate. To obtain the target of saponin constituents, the water layer was subjected to further isolation and purification [18, 19]. Briefly, the water layer was repeatedly separated by silica gel, Sephadex LH-20, and RP-C18 chromatography and yielded three new compounds (**1–3**) (Fig. 1). The structures of these metabolites were identified by IR, NMR (1D/2D), mass spectrometry, and electronic circular dichroism (ECD) analyses, as well as chemical reactivity.

**Fig. 2:**
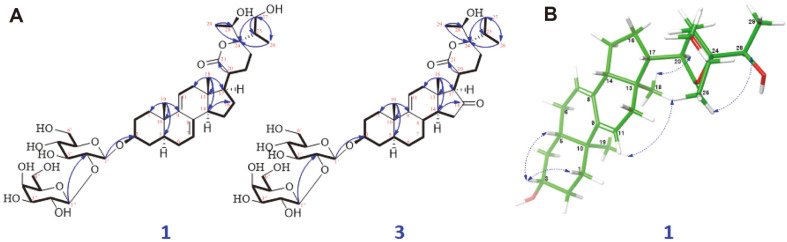
A. The key HMBC correlations (blue arrows) and COSY (bold), of saponins 1 and 3. B. Significant NOESY (→) correlations of aglycon of 1. The energy-minimized 3D of 1 was yielded by Macromodel (Version 12.5, Schrodinger LLC) program.

**Fig. 3:**
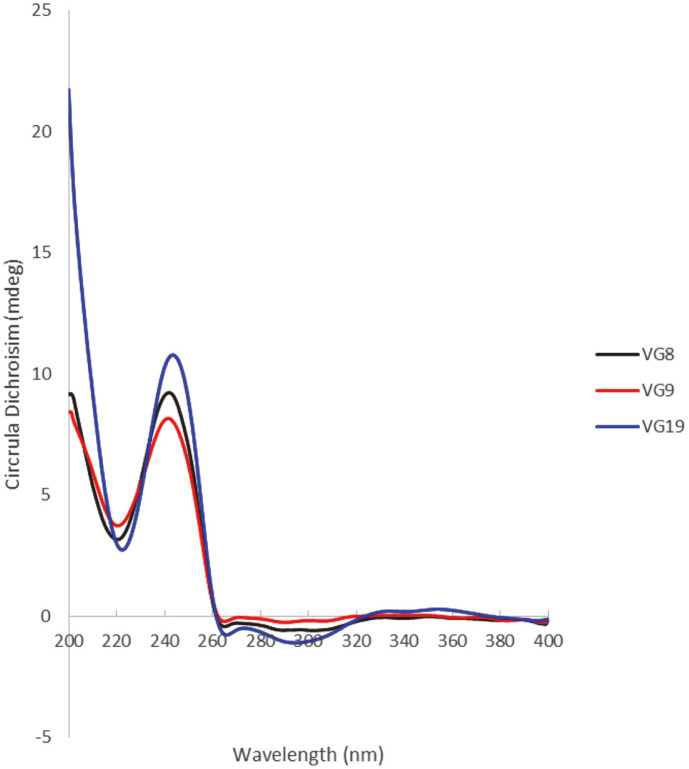
Experiment ECD spectra of compounds 1-3; Compound 1 (VG19), compound 2 (VG9), and compound 3 (VG8), respectively.

Substance **1** was yielded as a white amorphous powder with the molecular formula C_41_H_64_O_15_, which was identified from its HR-ESI-MS at *m/z* [M+Cl]^−^ 831.3892; (calcd for C_41_H_64_ClO_15_^-^, 831.3939). The NMR data of **1** were determined based on 1D, 2D NMR and ECD analyses, and by comparison with previously reported vernocuminosides [20]. These data (Table 1) showed that **1** is a Δ^7,9(11)^ stigmastane-type steroid saponin with a δ-lactone ring system. Indeed, the ^1^H NMR representation of **1** displayed signals of two olefinic protons [δ_H_ 5.43 (^1^H, s, H-7) and 5.50 (^1^H, brd, J = 5.5 Hz, H-11)], a distinctive H-3 multiplet [δ_H_ 3.72 (^1^H, m, H-3), two angular methyls [δ_H_ 0.66 (3H, s, H-18) and 0.94 (3H, s, H-19)], a propanyl-1-ol unit [δ_H_ 2.12 (^1^H, m, H-25), 1.06 (3H, d, J = 7.0 Hz, H-26), and 3.45 (2H, m, H-27)], and another doublet methyl [δ_H_ 1.26 (3H, d, J = 6.5 Hz, H-29)]. In addition, two sets of proton signals for glucopyranosyl and galactopyranosyl units, along with their anomeric protons [δ_H_ 4.55 (^1^H, d, J = 7.5 Hz, H-1¢) and 4.51 (^1^H, d, J = 8.0 Hz, H-1¢¢), were shown in the ^1^H NMR spectrum. The large coupling constants (J = 7.5 Hz between H-1¢ and H-2¢, J = 8.0 Hz between H-1¢¢ and H-2¢¢) supported β-linkage of the sugar moieties. The ^13^C NMR data of 1 revealed 42 carbon resonances, containing 29 for the aglycone moiety and 12 for the two sugar units. The ^13^C NMR data showed the existence of a carbonyl [δ_C_ 177.4 (C-21), four olefinic carbons [δ_C_ 121.7 (C-7), 137.2 (C-8), 145.4 (C-9), 119.4 (C-11)], two oxygenated methine carbons [δ_C_ 79.9 (C-3), 71.1 (C-28), and four methyl carbons [δ_C_ 11.9 (C-18), 19.9 (C-19), 12.6 (C-26), 17.6 (C-29)] for the aglycone moiety. The existence of the δ-lactone unit in the side chain of **1** was deduced by the connectivities of H-20/H-22/H-23, H-25/H-26/H-27, and H-28/H-29, together with the heteronuclear multiple bond Correlation (HMBC) correlations between H-22 and C-21/C-24, H-26 and C-24/C-25/C-27, and H-29 and C-24/C-28. The HMBC correlations from H-17 to C-20/C-21 allowed us to determine the location of the δ-lactone unit at C-17 of the aglycone of 1. The HMBC from H-1¢ (δ_H_ 4.55) to C-3 (δ_C_ 79.9) demonstrated that the β-D-glucosyl group was connected to C-3. The position of the galactopyranosyl moiety at C-2¢ was estimated from a downfield shift of C-2¢ (δ_C_ 83.6) in **1** compared to C-2¢ (δ_C_ 75.1) of glucose in vernocuminoside H, as well as the long-range HMBC correlation of H-1¢¢ (δ_H_ 4.51) with C-2¢ (δ_C_ 83.7). The comparison between the NMR values of 1 and the reported NMR data showed that **1** has similar NMR values to vernocuminoside I (Ver I), which was recently purified from the stem bark *V. cumingiana* Benth [20]. The main difference is the replacement of an oxygenated methylene group in **1** by a methyl group in Ver I. This was also confirmed by the HMBC correlations of H-27 with C-24, C-25, and C-26. Thus, the planar structure of **1** was deduced. The stereochemistry of **1** was defined based on Nuclear Overhauser Effect Spectroscopy (NOESY) correlations. In particular, the NOESY cross peaks H-3/H-5, H-14/H-17, and H-18/H-19 indicated that A/B and C/D fused in *trans*, H-18 and H-19 had β-configurations, and H-3, H-5, and H-17 had α-configurations. In addition, the NOESY correlation of H-18/H-20 showed the relationship between the lactone ring E and β-orientation of H-20 showed the relationship between the lactone ring E and the positioning of H-20 in a β-structure. The stereochemistry of C-24 and C-28 (Fig. 2) was determined from the NOESY cross-peaks from H-26 to H-28. The stereochemistry of C-24 was deduced based on the ECD spectrum. The ECD spectrum of **1** showed the opposite signals to those of vernocuminoside H, a new saponin reported from the *Vernonia* genus [20]. Indeed, the circular dichroism spectrum of **1** showed λ_max_ (mdeg) 221 (+2.82), and 243 (+10.78) nm (Fig. 3). Thus, the absolute configuration of C-24 was assigned as S-form based on the established correlation between the absolute configuration and the Cotton effect's sign. Moreover, the stereochemistry of C-28 in **1** was elucidated as *R*-form, which was also suggested by consideration of the biosynthetic pathway [20]. Finally, the identification of the sugar residues as D-glucose and D-galactose was established through the absolute configurations obtained from the acid hydrolysis of **1**. This was further confirmed by comparing the results with authentic samples through TLC analysis (Acid hydrolysis and sugar identification section). As a result, the substance **1** was found to be a new compound, and was named vernogratioside A.

Substance **2** was yielded as a white amorphous powder with the molecular formula C41H64O14, as determined from the HR-ESI-MS at *m/z* 779.4212 [M-H]^−^ (calcd for C41H63O14-, 779.4223). The NMR data for the substance were comparable to those of **1**, with the exception of the missing the oxygenated methylene at C-27 in **1** (which was replaced by a methyl group in **2**); also, the C-27 chemical shift was more upfield (δ_C_ 17.6) compared to δ_C_ 63.9 in **1**. Additionally, the NMR data of **2** is similar to those of Ver I, the only difference is only the configuration C-24 position. This was confirmed from the HMBC correlations of H-27 with C-24, C-25, and C-26. The relative configuration of **2** was established from the NOESY correlations, as described for **1**. The ECD spectra obtained through experimentation of **2** and **1** displayed identical Cotton effects (as shown in Fig. 3), which enabled the determination of the absolute configuration of C-24 and C-28 in **2** as 24*S*, and 28*R*, respectively. Thus, the chemical structure of **2** was identified as shown in Fig. 1, and was named vernogratioside B.

Substance **3** was yielded as a white amorphous powder with the molecular formula C_41_H_64_O_15_, as verified by the ion peak at *m/z* 797.4316 [M+H]^+^ (calcd for C_41_H_65_O_15_^+^ 797.4318) in the HR-ESI-MS spectrum. Comparison of the ^1^H and ^13^C NMR data of **3** with those of **2** indicated that **3** has similar NMR values to **2**, except for the absence of two olefinic carbon signals at C-7 and C-8. This was demonstrated by the upfield chemical shifts of C-7 (δ_C_ 26.6) and C-8 (δ_C_ 54.2) compared to δ_C_ 121.6 and 137.3 in **2**, respectively. In addition, based on the agreement in the NMR data of **3** and **2**, the downfield shift at C-16 (δ_C_ 212.2) in **3** with the missing carbon signal of the methylene group indicated that there was a ketone group at C-16 in **3**, instead of a methylene group in **2**. This was determined by the HMBC correlations of H-14 (δ_H_ 2.12)/H-15 (δ_H_ 2.11, 2.60) and C-16 (δ_C_ 212.2). The stereochemistry of **3** was the same as those of **2**, based on the NOESY and ECD spectra. The structure of **3** was thus established as shown in Fig. 1 and designated as vernogratioside C.

### The α-Glucosidase Inhibitory Activities of Compound 2 *in silico* and In Vitro

**Fig. 4:**
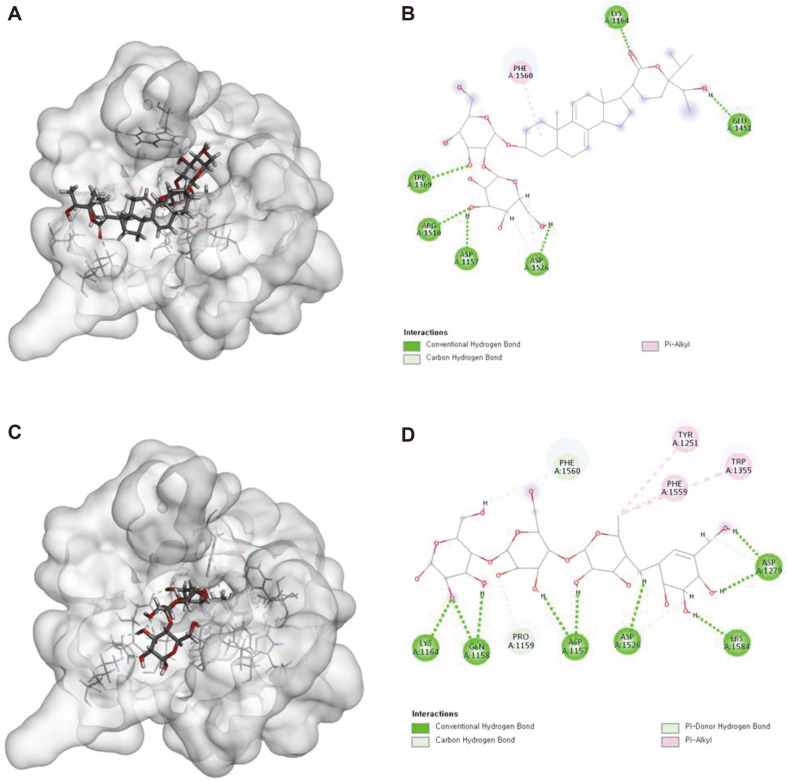
Docking simulation of the interactions from compound 2 (A), (B), and positive control (acarbose) (C), (D) to CtMGAM, 2D and 3D, respectively.

**Fig. 5:**
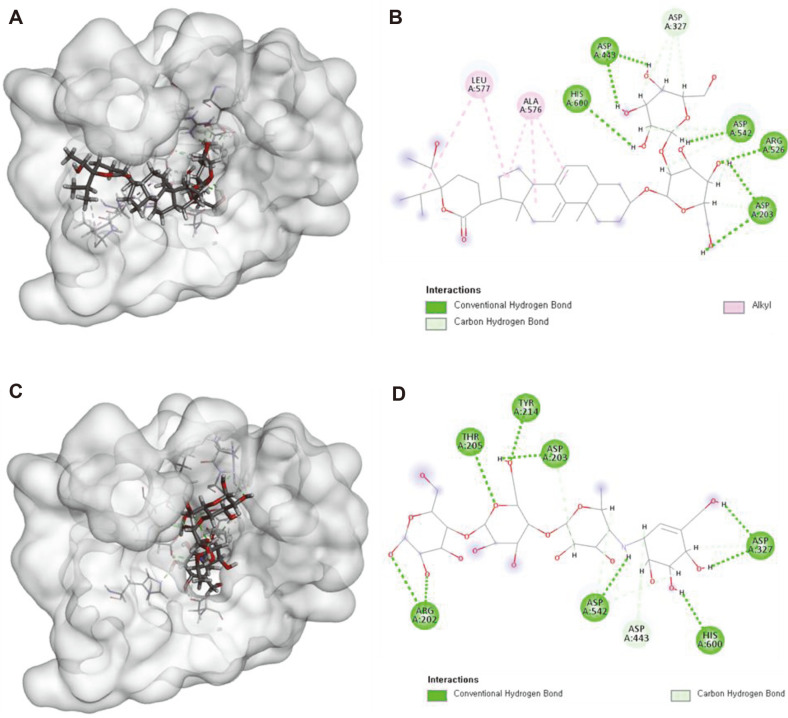
Docking simulation of the interactions from compound 2 (A), (B), and positive control (acarbose) (C), (D), to NtMGAM, 2D and 3D, respectively.

Alpha-glucosidase inhibitory activity refers to the ability of a substance to reduce the activity of the alpha-glucosidase enzyme [7]. This enzyme is responsible for breaking down complex sugars into simpler forms that can be absorbed by the body. Inhibiting the action of alpha-glucosidase can lead to a decrease in the rate of sugar absorption and a lowering of blood sugar levels, which is why substances with alpha-glucosidase inhibitory activity are of interest as potential treatments for conditions such as type 2 diabetes [5]. Compound **2** obtained the highest amount of three new compounds (Please see the purification of compounds section). We assumed that **2** was present in the most significant amounts in *V. gratiosa*. Therefore, it was selected for further *in silico* and in vitro experiments of α-glucosidase inhibitory activities. Molecular docking analysis was performed for two types of human MGAM to evaluate the α-glucosidase inhibitory activities of stigmastane-type steroidal saponins (NtMGAM and CtMGAM) [21]. NtMGAM and CtMGAM have the same amino- and carboxy-terminal catalytic domains, but different substrate specificities; NtMGAM shows a preference for shorter α-(1,4) oligosaccharide units as substrates, whereas CtMGAM shows a preference for longer chains [22]. The total score is a critical aspect of molecular docking as it helps to predict the most energetically favorable binding mode between the ligand and receptor. The lower the total score, the stronger the predicted binding affinity between the ligand and receptor. Compared to the positive control, acarbose, compound **2** had significantly different docking scores for both CtMGAM 3TOP and NtMGAM 2QMJ (6.55 and 8.59, respectively), as indicated in Table 2. As shown in Figs. 4 and 5, and Table 2, this interaction between compound **2** and CtMGAM required eight hydrogen bonds with six amino acid, including Asp1157 (1.76 Å), Lys1164 (2.01 Å), Trp1369 (1.84 Å), Glu1451 (1.85 Å), Arg1510 (1.89 Å), and Asp1526 (2.20, 2.42, 2.86 Å). Fig. 4B also shows one hydrophobic bond with Phe1560, which is a Pi-alkyl interaction with the cyclohexane ring of **2** with a distance of 4.53 Å. In addition, this interaction between **2** and NtMGAM required sixteen hydrogen bonds with six amino acids, including Asp203 (1.74, 2.01, 2.98 Å), Asp327 (2.24, 2.52, 2.85 Å), Asp443 (1.72, 1.92 Å), Arg526 (1.91, 2.23 Å), Asp542 (2.15, 2.28, 2.35, 3.00, 3.08 Å), and His600 (2.16 Å). Fig. 5B also shows five hydrophobic bonds with Ala576 and Leu577, which are alkyl interactions with the cyclohexane ring of **2** with a distance of 3.66, 4.65, 4.85, 5.19, and 5.04 Å, respectively. Interestingly, compared to the positive control, acarbose, Asp1157 and Asp1526 for CtMGAM, and Asp203, Asp327, Asp443, Asp542, and His600 for NtMGAM, were found to have important roles in inducing and stabilizing the active conformations of the respective proteins. It was also found that these catalyst residues play a partly important role in the formation of hydrogen interactions during molecular docking. These findings suggested that compound **2** may be introduced into the active site of the enzyme and bind tightly to the catalytic amino acid residues via a variety of interactions, thereby inhibiting the α-glucosidase activity. Compounds **1-3** shared similar structures, which can provide information for the in vitro and *in silico* of a-glucosidase inhibitory activity by comparing the structure-activity relationship. Thus, the docking results showed that stigmastane-type steroidal glycosides are promising candidates as antidiabetic agents.

To evaluate its potential antidiabetic effects, the ability of to inhibit alpha-glucosidase activity of compound **2** was evaluated. The results showed that compound **2** exhibited inhibitory activity against α-glucosidase at a concentration of 500 μg/ml in comparison to acarbose as a positive control (58.66 ± 1.07 vs. 81.70 ± 1.53 %). These results suggested that stigmastane-type steroidal saponins from *V. gratiosa* are promising candidates as antidiabetic agents. Further in vitro and in vivo studies of (**1–3**) are required to confirm the potential antidiabetic effects of steroidal saponins from *V. gratiosa*. In our continuous efforts to identify active components from medicinal plants, this study not only contributes to the diversity of chemical components but also provides the potential α-glucosidase inhibitory activity of stigmastane-type steroidal saponins from *V. gratiosa*.

## Supplemental Materials

Supplementary data for this paper are available on-line only at http://jmb.or.kr.

## Figures and Tables

**Table 1:** ^1^H and ^13^C-NMR data of compounds 1-3.

No	**1**		**2**		**3**	

	δ_C_^a,b^	δ_H_^a,c^ (mult. J = Hz)	δ_C_^a,b^	δ_H_^a,c^ (mult. J = Hz)	δ_C_^a,b^	δ_H_^a,c^ (mult. J = Hz)
1	36.0	1.33 (m), 2.00 (m)	36.0	1.36 (m), 2.00 (m)	35.4	1.48 (m), 1.85 (m)
2	30.6	1.61 (m), 2.02 (m)	30.6	1.60 (m), 2.02 (m)	30.2	1.70 (m), 2.09 (m)
3	79.9	3.72 (m)	79.9	3.72 (m)	79.3	3.73 (m)
4	35.1	1.40 (m), 1.90 (m)	35.1	1.39(m), 1.90 (m)	36.1	1.43 (m), 1.82 (m)
5	40.6	1.39 (m)	40.6	1.40 (m)	44.0	1.48 (m)
6	31.0	1.96 (m)	31.0	1.95 (m)	27.0	1.21 (m), 2.02 (m)
7	121.7	5.43 (s)	121.6	5.43 (s)	26.6	1.49 (m), 1.84 (m)
8	137.2		137.3	-	54.2	3.06 (d, 10.0)
9	145.4		145.4	-	145.3	
10	37.1		37.1	-	39.1	
11	119.4	5.50 (d, 6.5)	119.5	5.52 (d, 6.5)	119.8	5.46 (m)
12	41.0	1.96 (m), 2.23 (m)	41.2	2.02 (m), 2.24 (m)	39.6	1.84 (m), 2.17 (m)
13	43.2		43.6	-	42.5	
14	52.8	1.15 (m)	52.6	2.23 (m)	49.7	2.12 (m)
15	23.7	1.81 (m), 1.48 (m)	23.7	1.15 (m), 1.64 (m)	46.1	2.11 (m), 2.60 (m)
16	27.4	1.98 (m), 1.50 (m)	26.6	1.52 (m), 1.92 (m)	212.2	
17	50.7	1.79 (m)	50.5	2.19 (m)	47.1	1.82 (m)
18	11.9	0.61 (s)	12.3	0.63 (s)	12.5	0.75 (s)
19	19.9	0.95 (s)	20.0	0.94 (s)	18.0	1.26 (s)
20	41.9	2.55 (m)	41.8	2.57 (m)	41.1	2.57 (m)
21	177.4		178.4		178.4	
22	27.4	1.14 (m), 1.55 (m)	23.7	1.84 (m), 2.10 (m)	23.9	1.65 (m), 2.10 (m)
23	23.5	1.60 (m)	23.0	1.85 (m), 2.05 (m)	23.0	1.85 (m), 2.04 (m)
24	91.1		91.1	-	91.2	
25	43.4	2.12 (m)	36.1	1.85 (m)	36.2	1.94 (m)
26	12.6	1.06 (s)	17.2	1.00 (d, 7.0)	17.2	1.00 (d, 7.0)
27	63.9	3.45 (m), 3.80 (m)	17.6	1.03 (d, 7.0)	17.6	1.03 (d, 7.0)
28	71.1	3.91 (m)	71.8	3.95 (q, 6.5)	71.9	3.95 (q, 6.5)
29	17.63	1.26 (d, 6.5)	17.9	1.19 (d, 6.5)	17.9	1.19 (d, 6.5)
30						
31						
1'	101.3	4.55 (d, 7.5)	101.4	4.56 (d, 7.5)	101.4	4.55 (d, 7.5)
2'	83.6	3.42 (m)	83.7	3.42 (dd, 7.5, 9.0)	83.7	3.41 (dd, 7.5, 9.0)
3'	77.7	3.29 (m)	77.7	3.29 (m)	77.8	3.29 (m)
4'	71.5	3.34 (m)	71.5	3.34 (m)	71.5	3.32 (m)
5'	77.8	3.60 (m)	77.7	3.58 (m)	77.9	3.58 (m)
6'	62.7	3.68 (m), 3.89 (m)	62.7	3.68 (dd, 5.5, 12.0)	62.7	3.67 (dd, 5.5, 12.0)
				3.87 (dd, 5.5, 12.0)		3.87 (m)
1''	106.2	4.51 (d, 8.0)	106.2	4.51 (d, 8.0)	106.3	4.51 (d, 8.0)
2''	73.5	3.64 (m)	73.5	3.64 (m)	73.7	3.62 (dd, 8.0, 9.5)
3''	74.7	3.53 (m)	74.7	3.53 (m)	74.7	3.52 (dd, 8.0, 9.0)
4''	70.0	3.89 (m)	70.0	3.88 (m)	70.1	3.87 (m)
5''	77.0	3.55 (m)	77.0	3.56 (m)	77.1	3.55 (m)
6''	62.1	3.77 (m)	62.1	3.77 (m)	62.3	3.77 (d, 6.5)

^a^Record in CD_3_OD ^b^125MHz ^c^500MHz; Assignments were elucidated based on COSY, HSQC, HMBC, and NOESY spectra

**Table 2:** Interaction and autodock score between α-glucosidase and compound 2.

CtMGAM_3TOP				NtMGAM_2QMJ

Sample	Total score^a^	Key residues	Total score^a^	Key residues
**2**	6.55	Asp1157(1.76), Lys1164(2.01), Trp1369(1.84), Glu1451(1.85), Arg1510(1.89), Asp1526(2.20, 2.42, 2.86)	8.59	Asp203(1.74, 2.01, 2.98), Asp327(2.24, 2.52, 2.85), Asp443(1.72, 1.92), Arg526(1.91, 2.23), Asp542(2.15, 2.28, 2.35, 3.00, 3.08), His600(2.16)
**Acarbose^b^**	13.81	Asp1157(2.03, 2.90), Gln1158(2.25, 2.29), Pro1159(2.37), Lys1164(1.94), Asp1279(1.95, 1.99, 2.85), Asp1526(2.23, 2.65), Phe1560(2.44), His1584(1.95)	14.28	Arg202(1.90, 2.31), Asp203(1.94, 2.80), Thr205(1.79, 2.90), Tyr214(2.09), Asp327(1.91, 1.97, 2.99) , Asp443(2.28, 2.92), Asp542(2.07, 2.56, 2.66), His600(2.04)

^a^Sanitize-Dock scores (total scores) were exhibited in −log10(*K*_d_)^2^ units used to identify binding affinities

^b^Positive control
