# Supplementary material for: Alpha-Glucosidase Inhibitory Activity of Saponins Isolated from Vernonia gratiosa Hance
Source: J Microbiol Biotechnol. 2023 Mar 6;33(6):797–805. doi: 10.4014/jmb.2212.12040 (PMC10331943; doi:10.4014/jmb.2212.12040)
Supplement: Supplementary file 1 [file jmb-33-6-797-supple.pdf]

J. Microbiol. Biotechnol.
https://doi.org/10.4014/jmb.2212.12040

# Supplementary Figures

## Alpha-Glucosidase Inhibitory Activity of Saponins Isolated from *Vernonia gratiosa* Hance

Pham Van Cong [a,b,*], Hoang Le Tuan Anh [a,b,✉], Le Ba Vinh [c,d,*], Yoo Kyong Han [c], Nguyen Quang Trung [a], Bui Quang Minh [a], Ngo Viet Duc [a], Tran Minh Ngoc [f], Nguyen Thi Thu Hien [g], Hoang Duc Manh [h], Le Thi Lien [k], and Ki Yong Lee [c,✉]

[a] *Center for Research and Technology Transfer, Vietnam Academy of Science and Technology (VAST), 18 Hoang Quoc Viet, Hanoi 100000, Vietnam*

[b] *Graduate University of Science and Technology, VAST, Hanoi, Vietnam*

[c] *College of Pharmacy, Korea University, Sejong 30019, Republic of Korea*

[d] *Institute of Marine Biochemistry, VAST, Hanoi, Vietnam*

[e] *National Institute of Medicinal Materials (NIMM), 3B Quang Trung, Hoan Kiem, Hanoi, Vietnam*

[f] *Vietnam Military Medical University, 160 Phung Hung, Ha Dong, Hanoi, Vietnam*

[g] *Hanoi University of Mining and Geology, Pho Vien, Duc Thang, Bac Tu Liem, Hanoi, Vietnam*

[h] *National Institute of Medicinal Materials, Vietnam*

[k] *Mientrung Institute for Scientific Research, VAST, Huynh Thuc Khang, Thua Thien Hue 52000, Vietnam*

[*] These authors contributed equally to this work

✉ Corresponding author's contact information:

Email contact: hltanh@ctctt.vast.vn (H.L.T. Anh)

Email contact: kylee11@korea.ac.kr (K.Y.Lee)

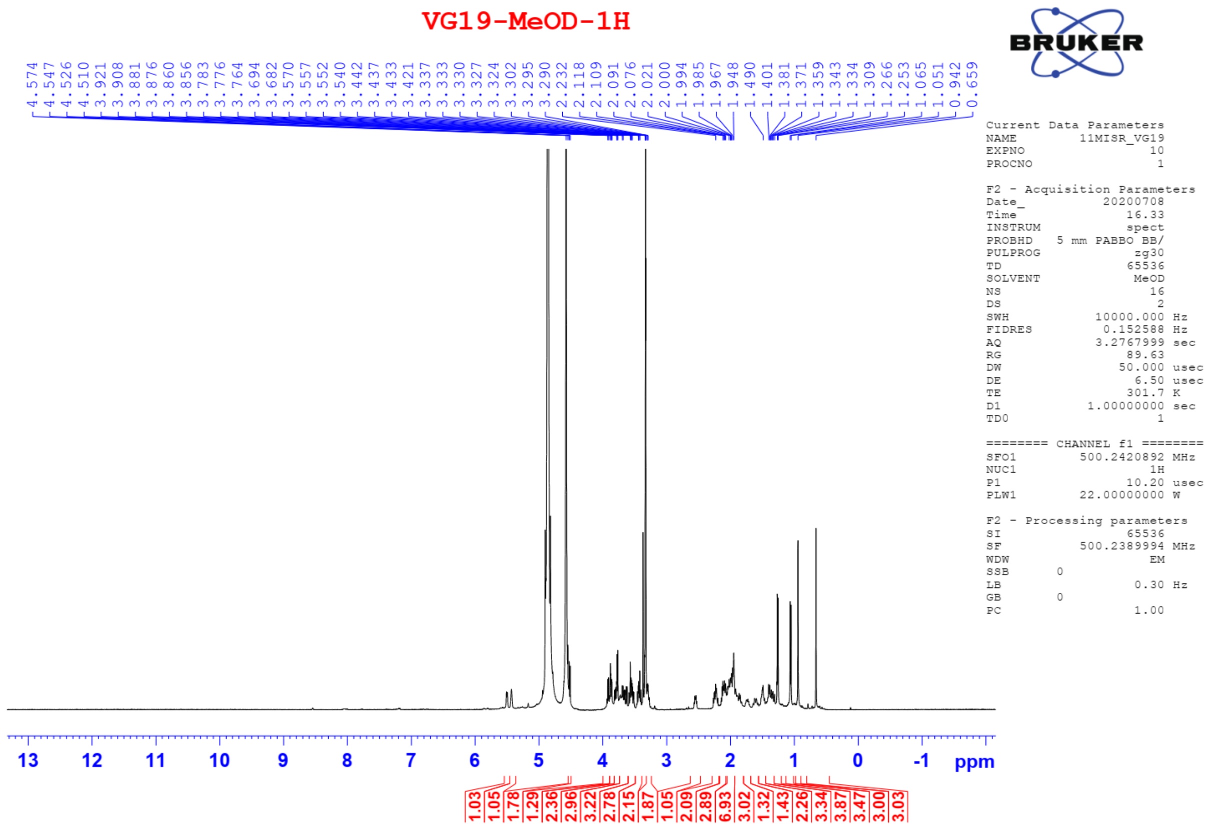

**Figure S 1.** $^1$H - NMR spectrum of compound **1** (500 MHz, MeOD)

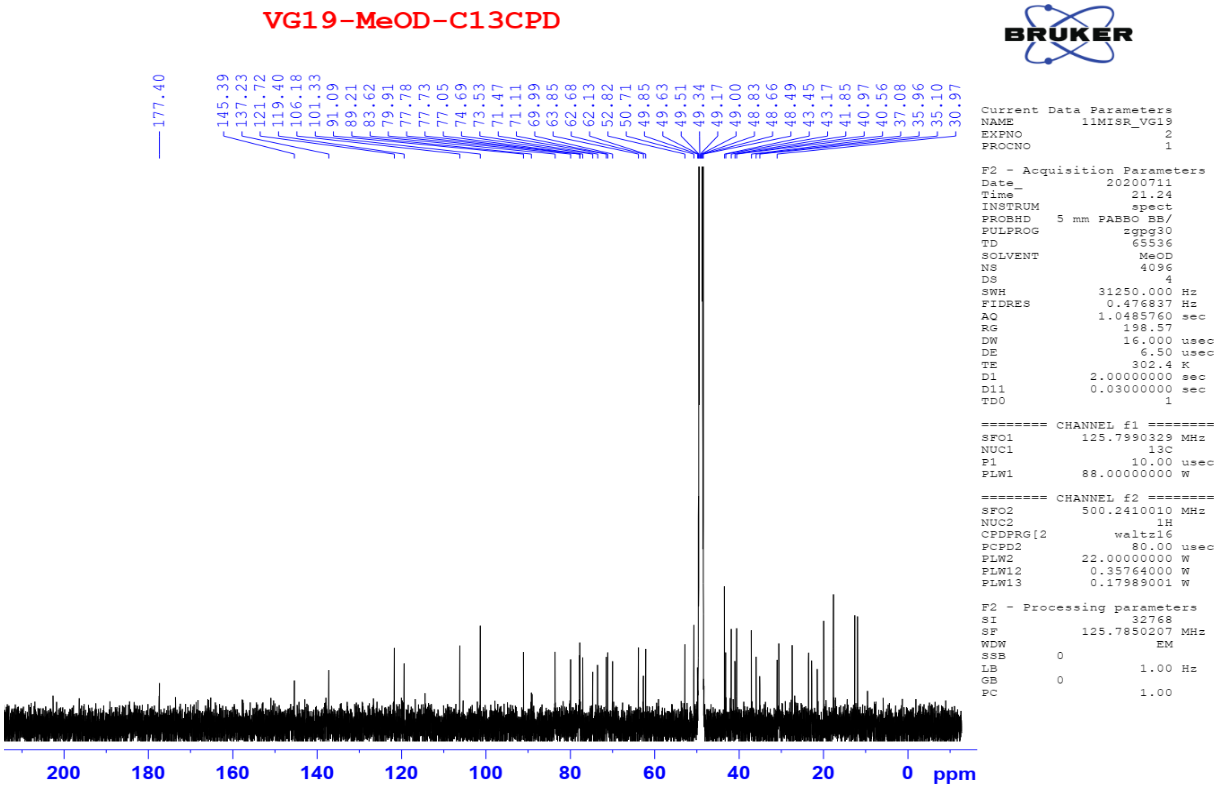

**Figure S 2.** $^{13}C$ - NMR spectrum of compound **1** (125 MHz, MeOD)

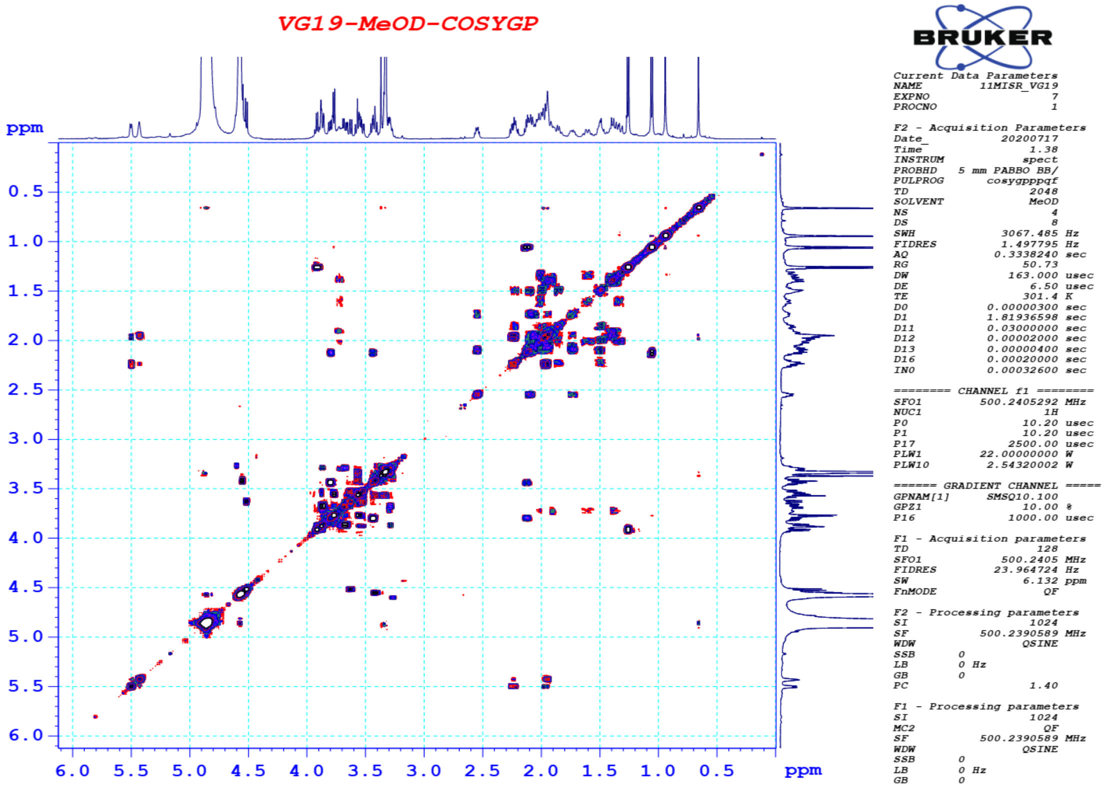


**Figure S 3.** $^{1}H$ - $^{1}H$ COSY spectrum of compound **1** (500/500 MHz, MeOD)

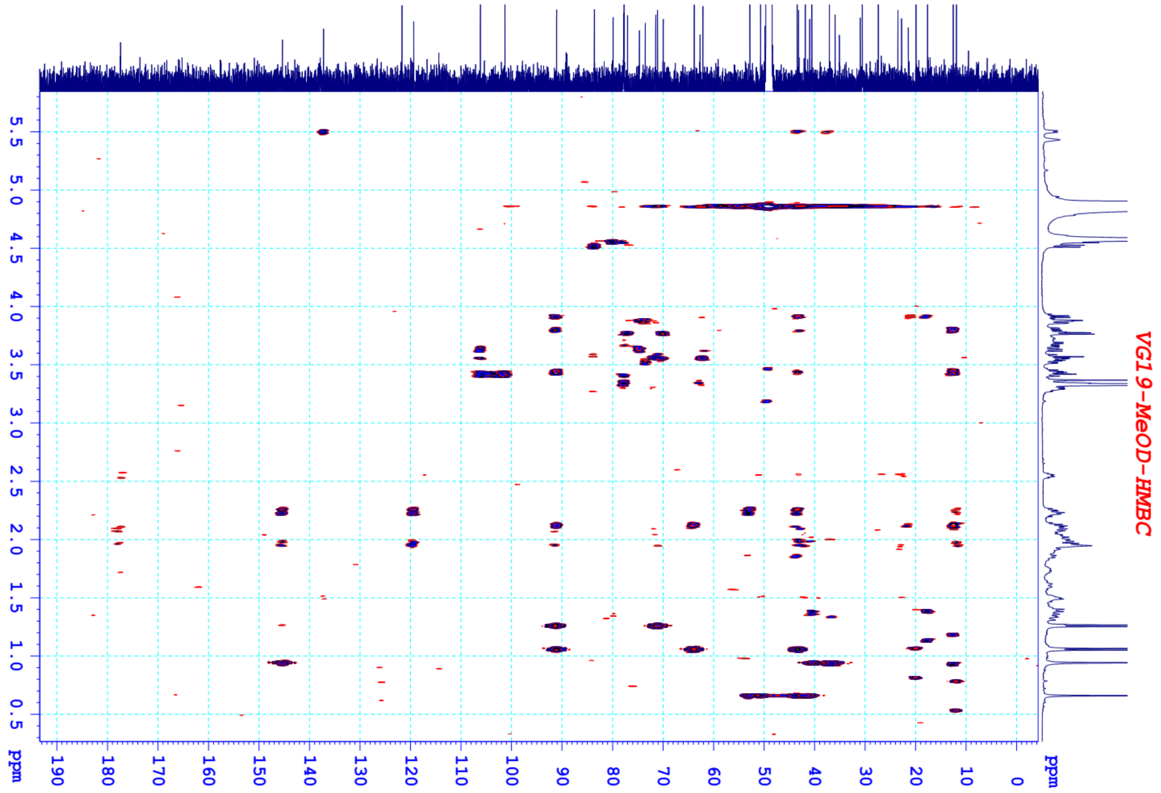


**Figure S 4.** HMBC spectrum of compound **1** (500/125 MHz, MeOD)

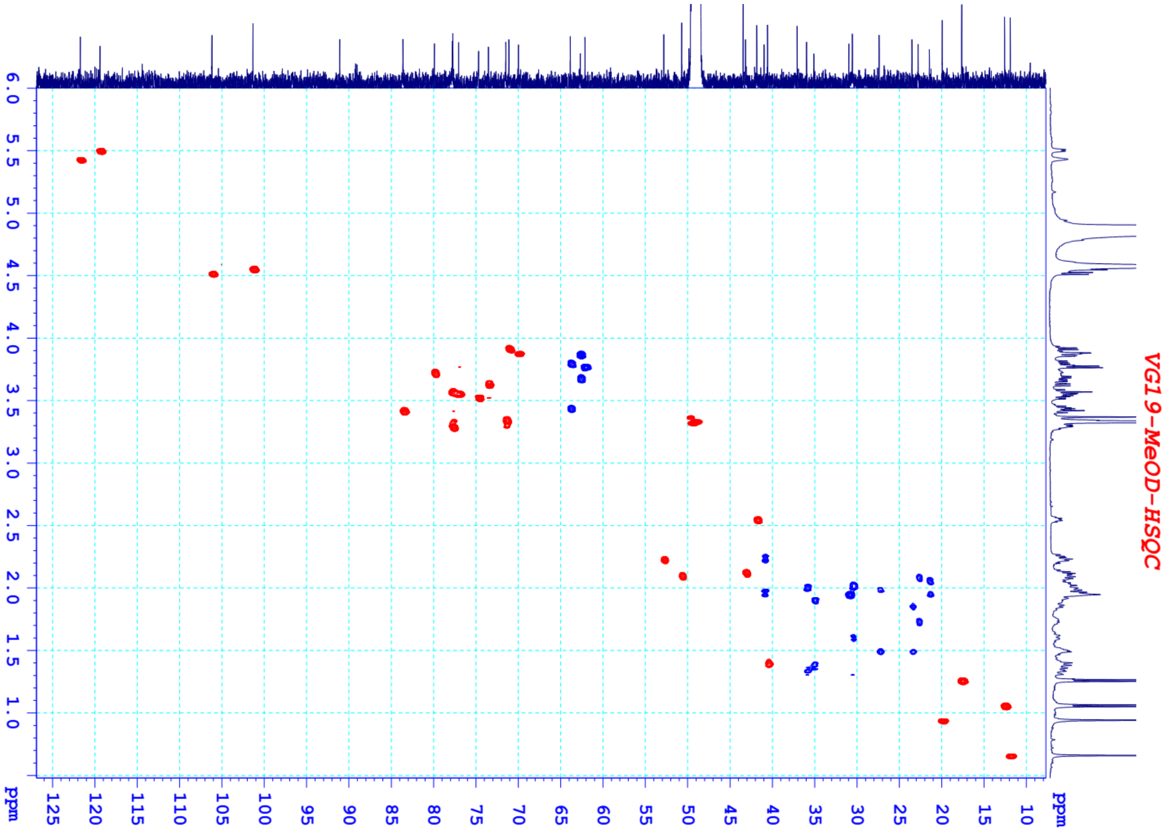


**Figure S 5.** HSQC spectrum of compound **1** (500/125 MHz, MeOD)

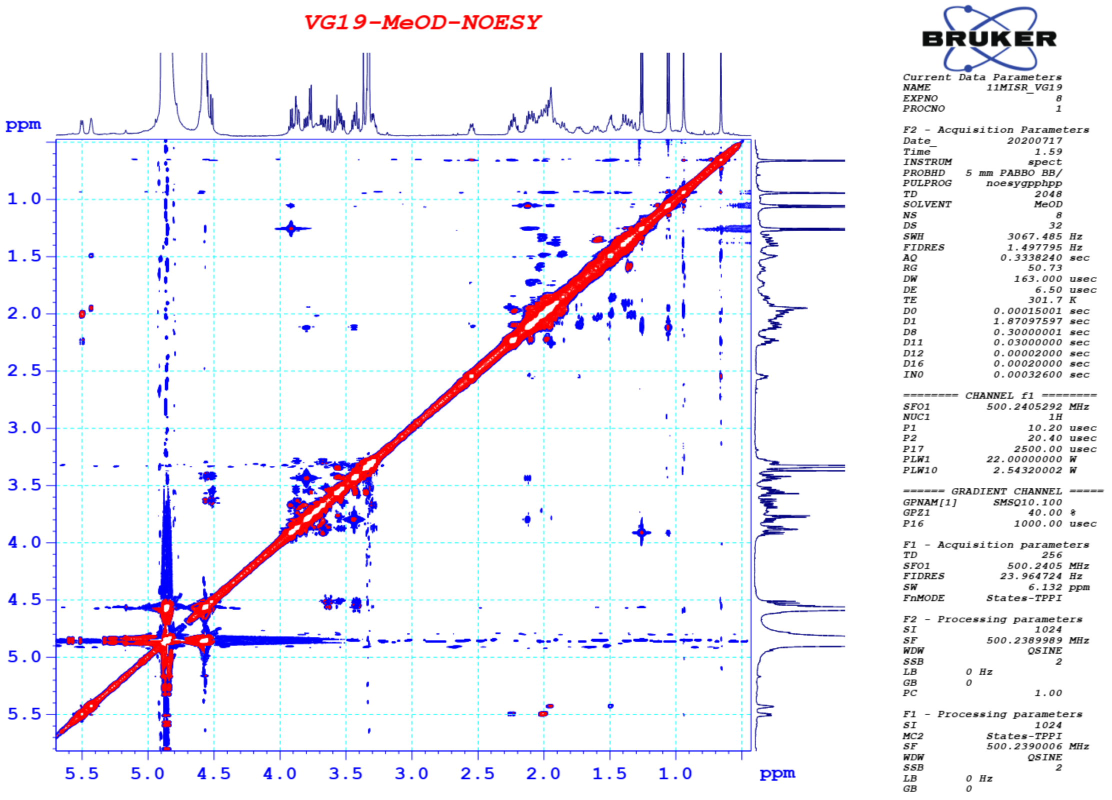


**Figure S 6.** NOESY spectrum of compound **1** (500/500 MHz, MeOD)

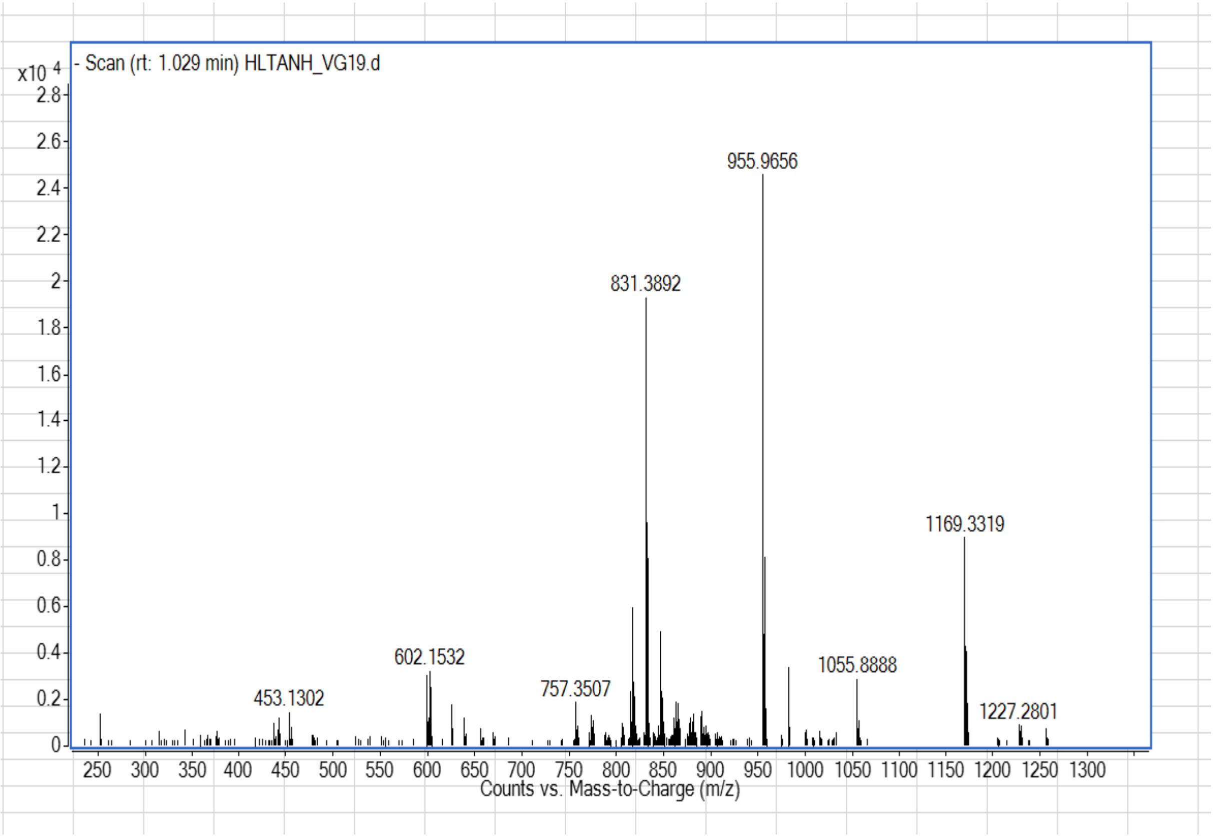


**Figure S 7.** HR-ESI-TOF-MS spectrum of compound **1**

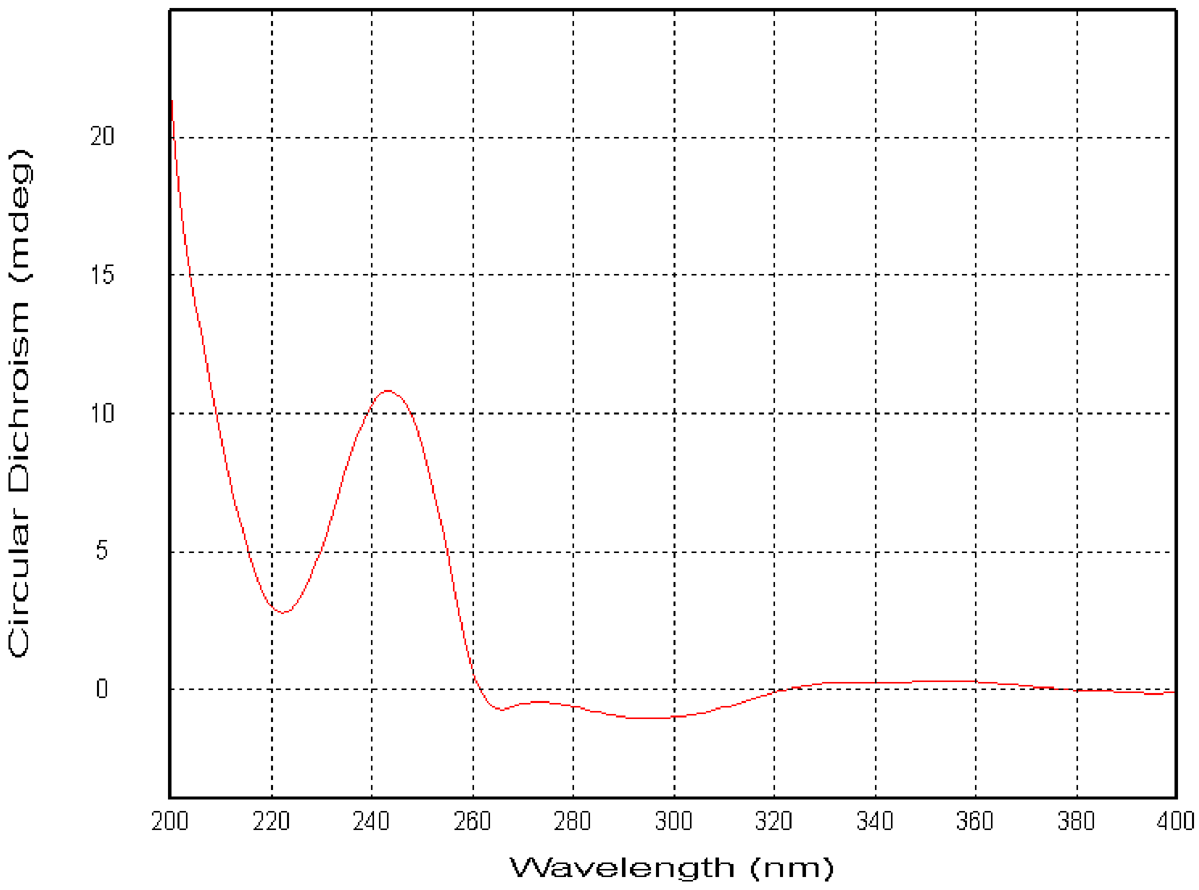


**Figure S 8.** CD spectrum of compound **1**

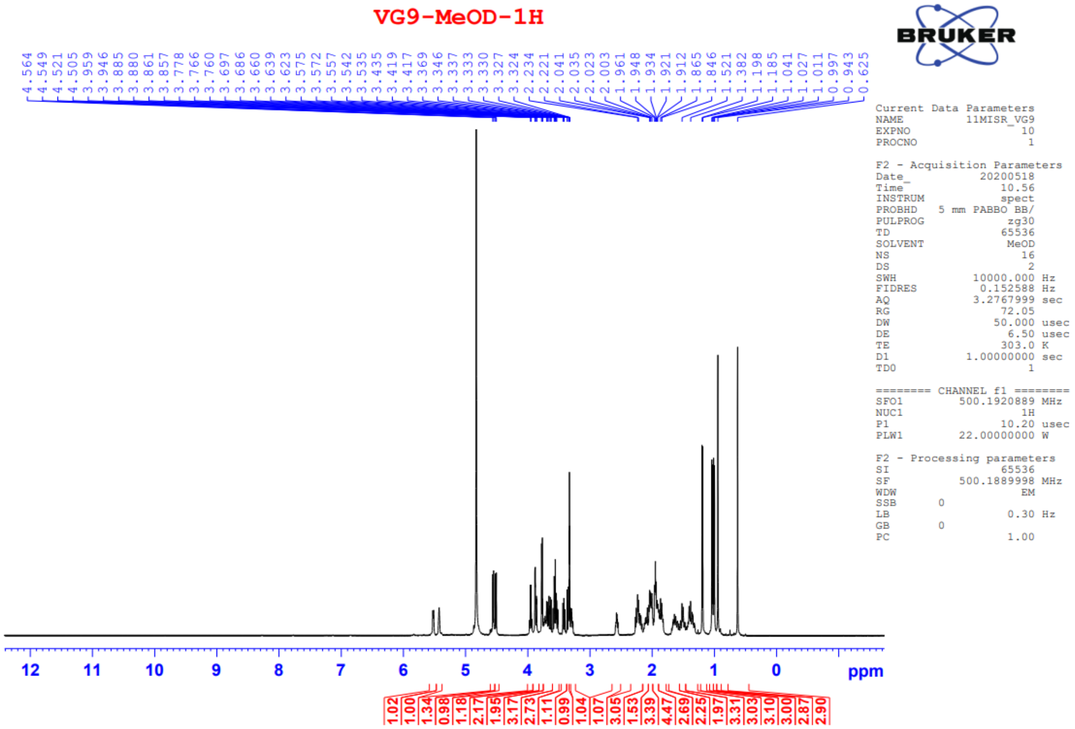


**Figure S 9.** $^{1}$H-NMR spectrum of compound **2** (500 MHz, MeOD)

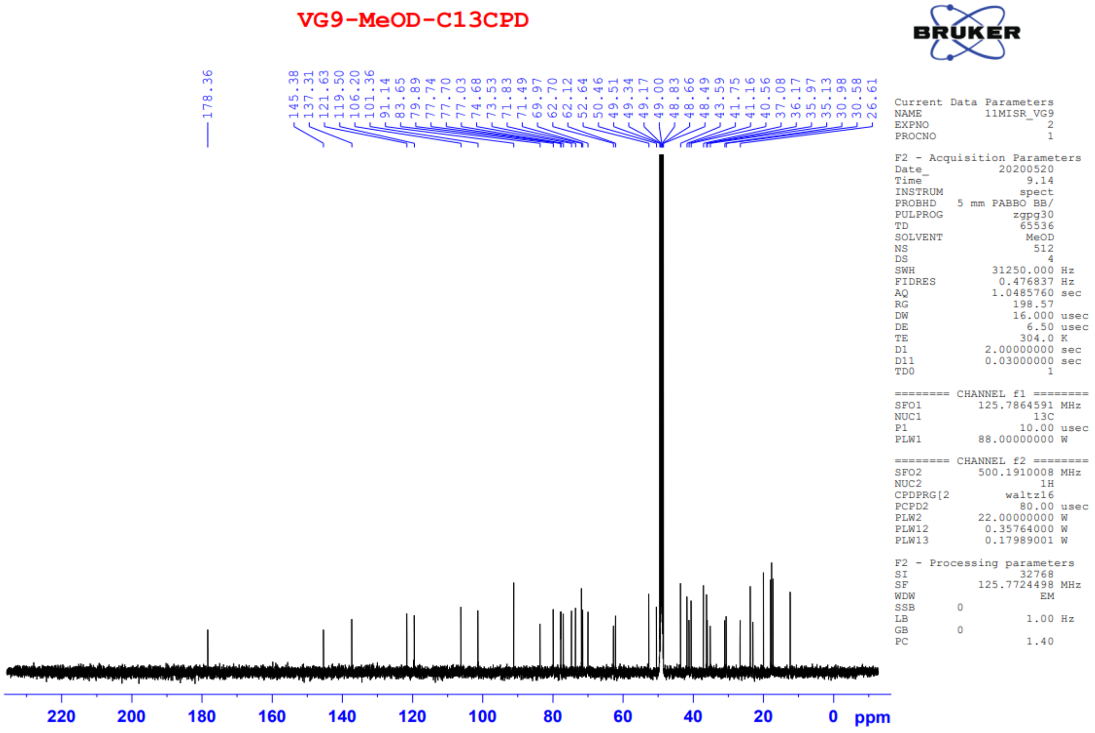


**Figure S 10.** $^{13}$C - NMR spectrum of compound **2** (125 MHz, MeOD)

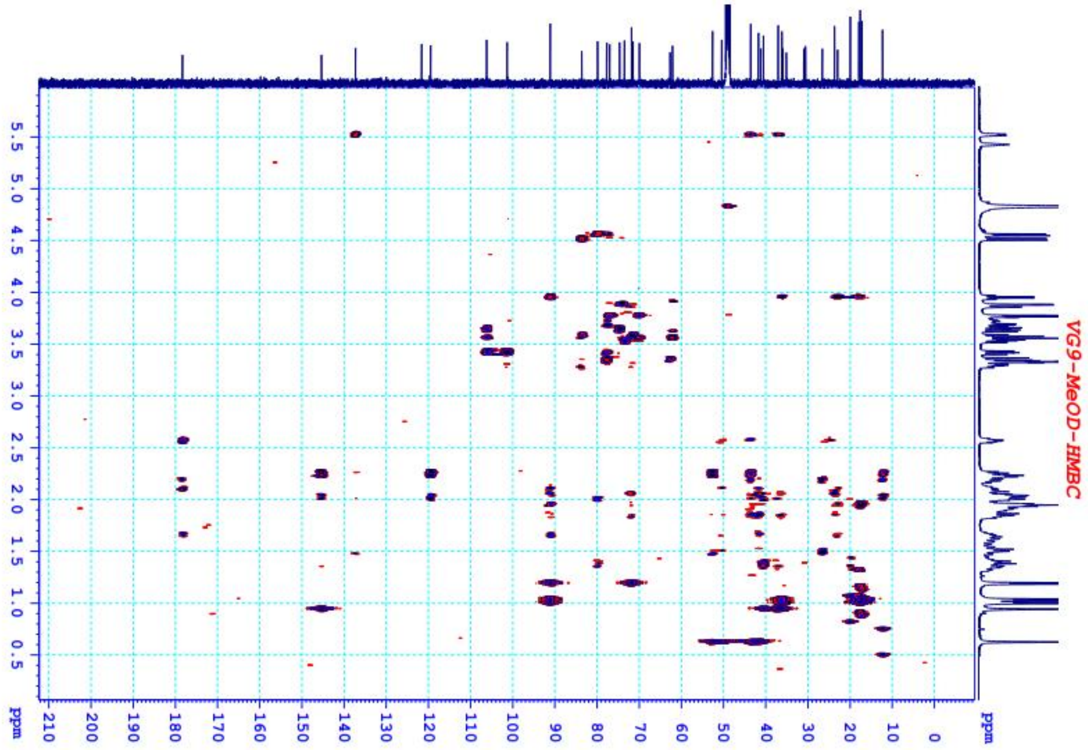


**Figure S 11.** HMBC spectrum of compound **2** (500/125 MHz, MeOD)

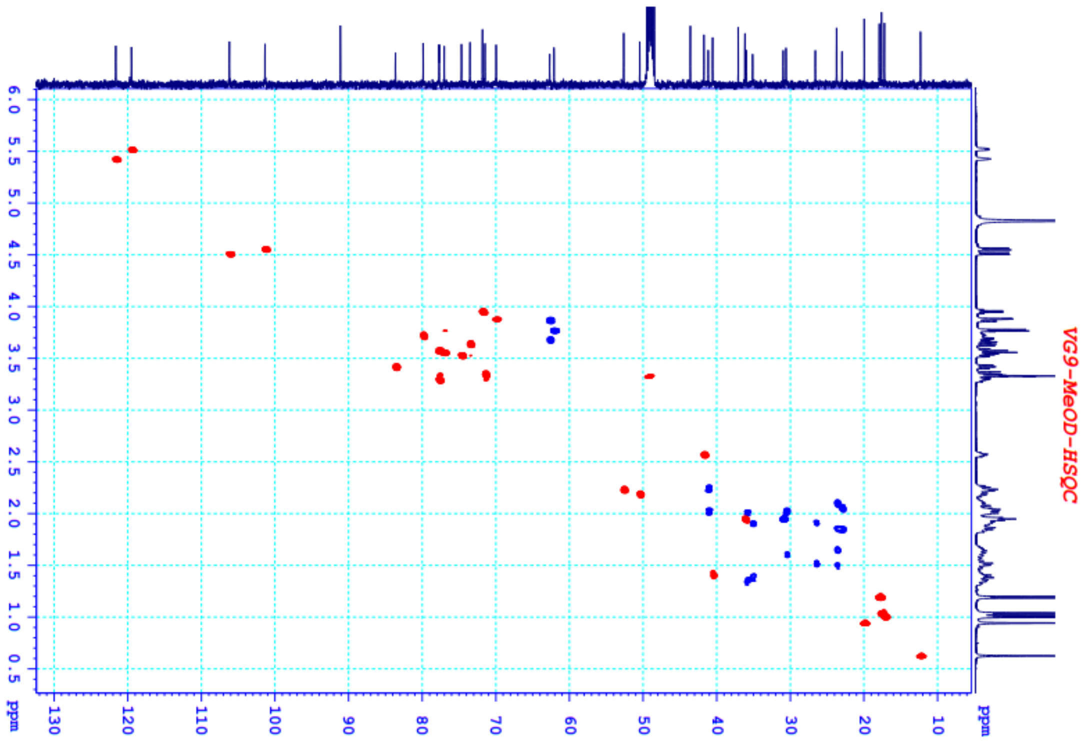


**Figure S 12.** HSQC spectrum of compound **2** (500/125 MHz, MeOD)

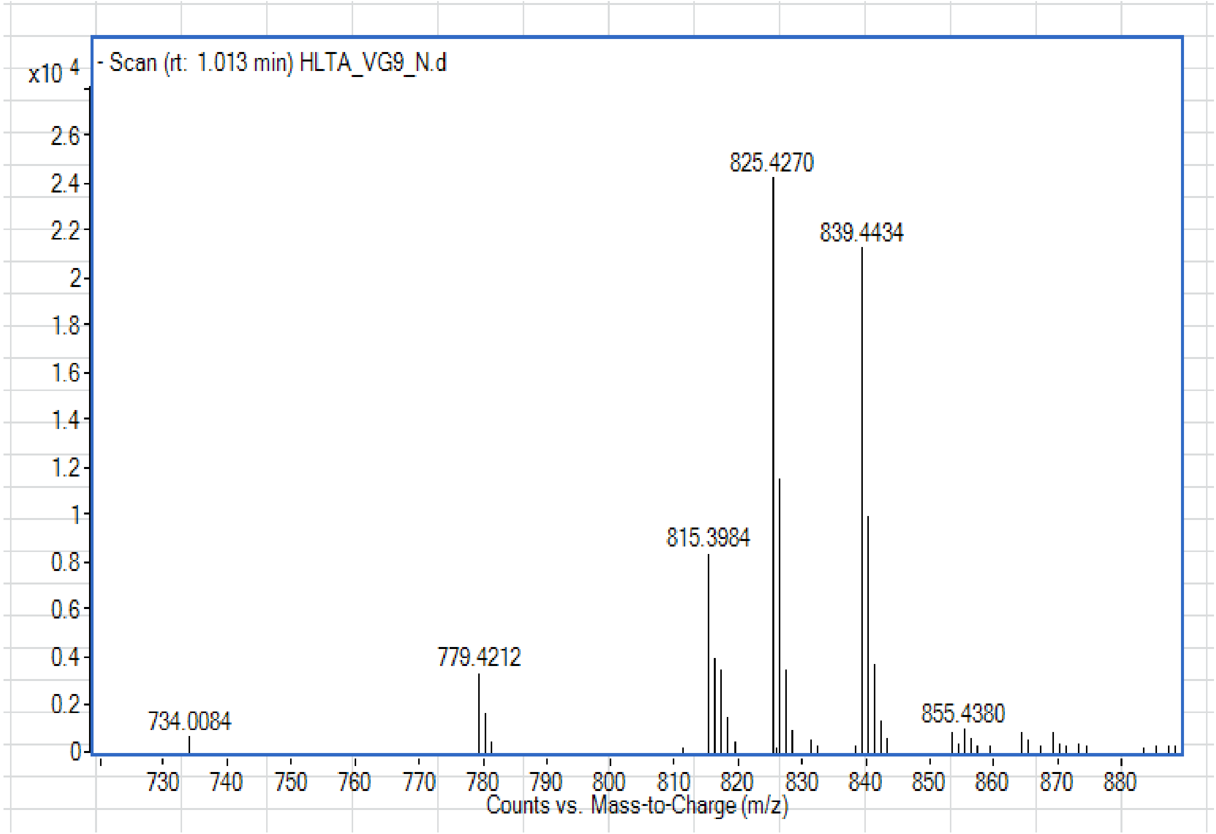


**Figure S 13.** HR-ESI-TOF-MS spectrum of compound **2**

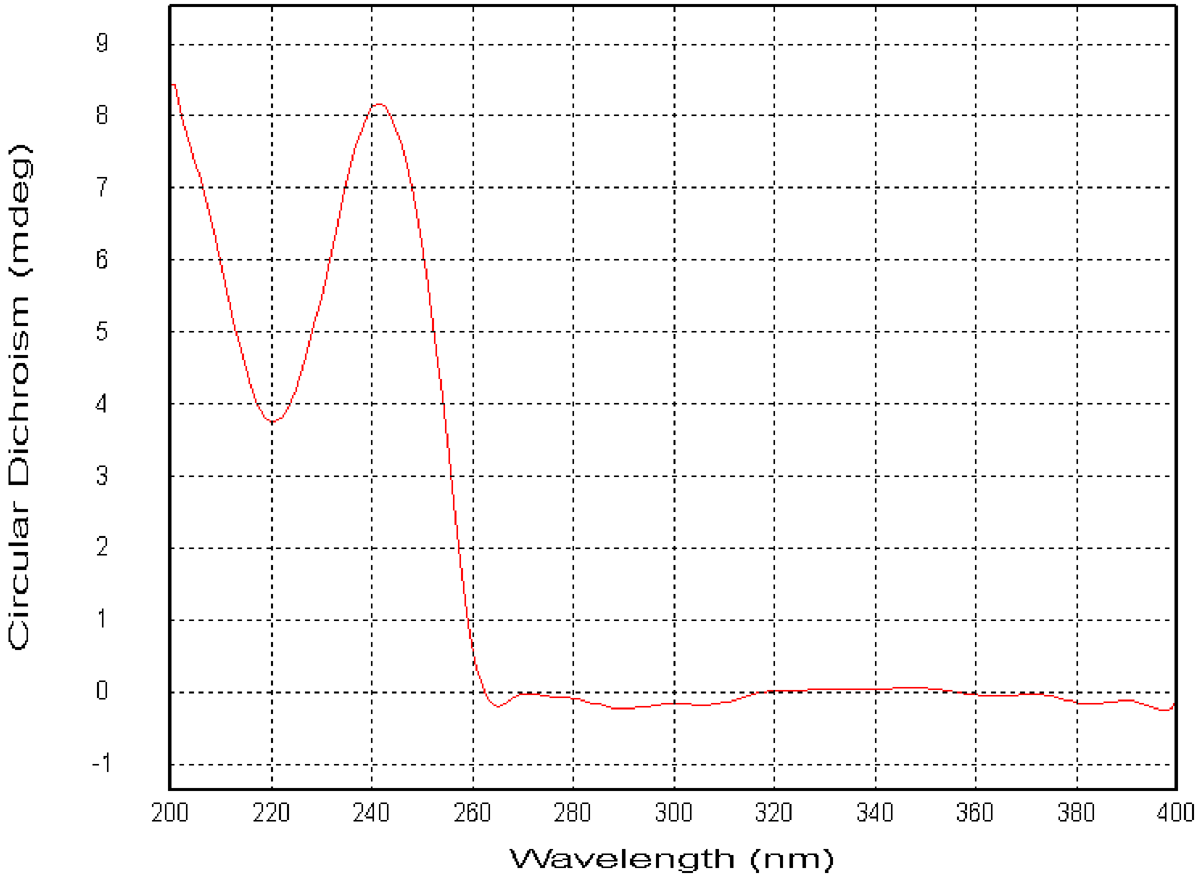


**Figure S 14.** CD spectrum of compound **2**

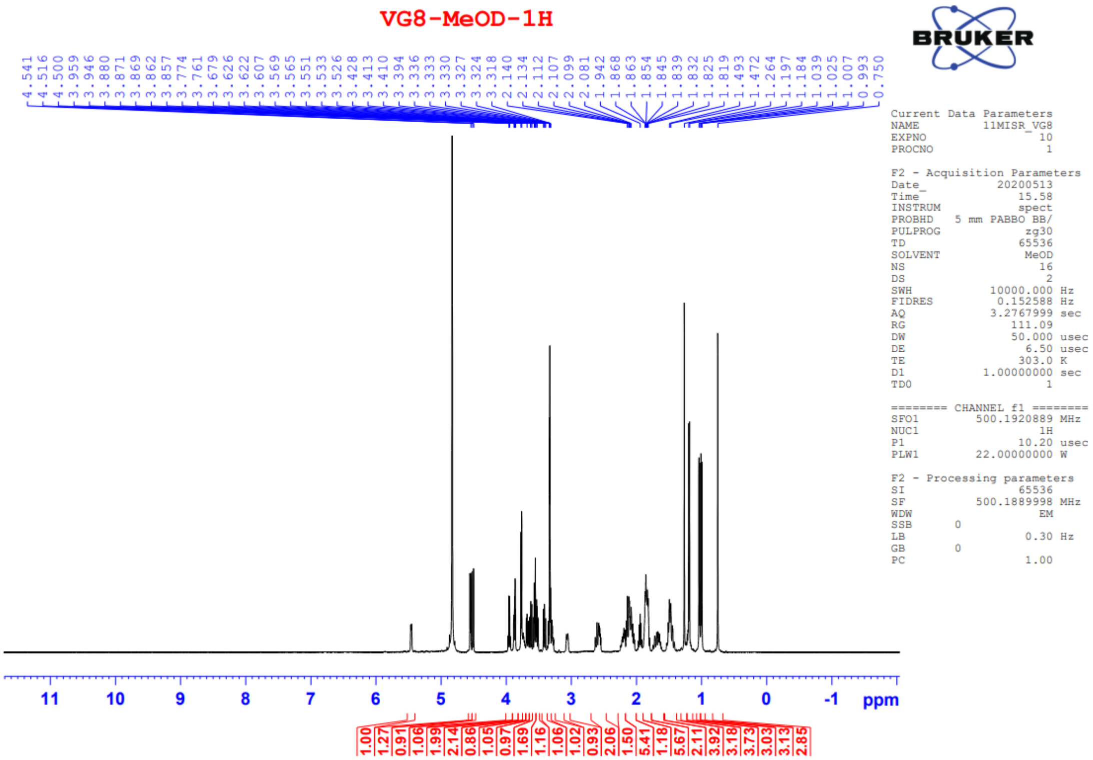


**Figure S15.** $^1$H-NMR spectrum of compound **3** (500 MHz, MeOD)

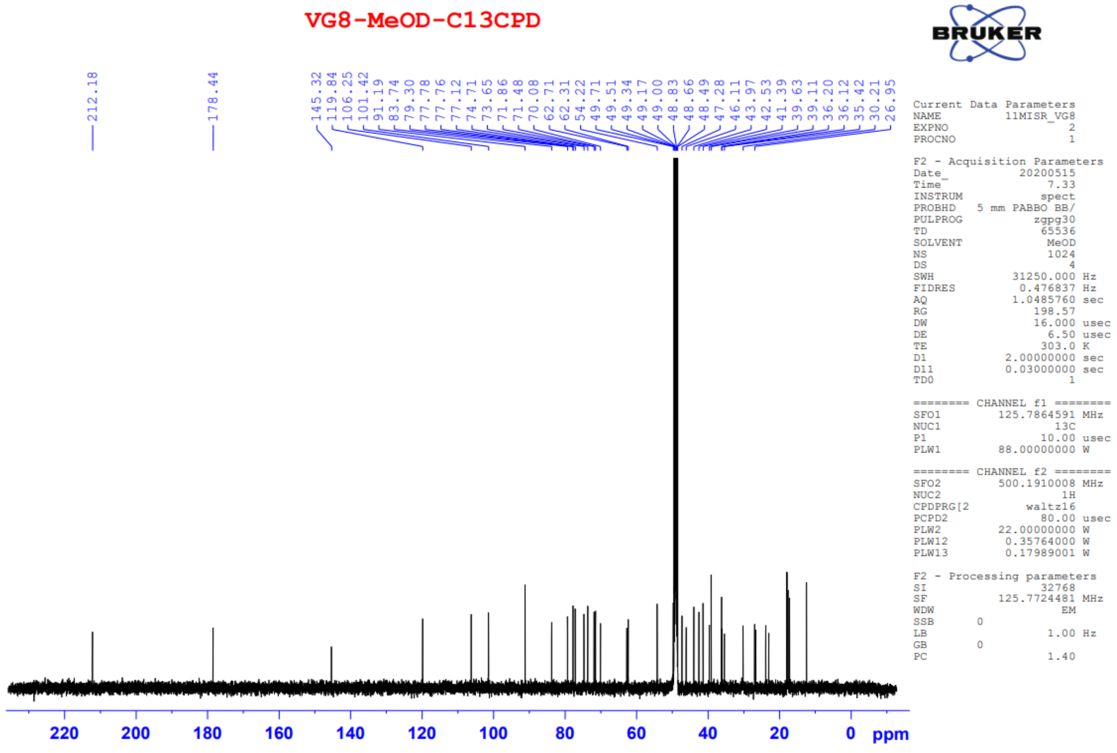


**Figure S16.** $^{13}$C-NMR spectrum of compound **3** (125 MHz, MeOD)

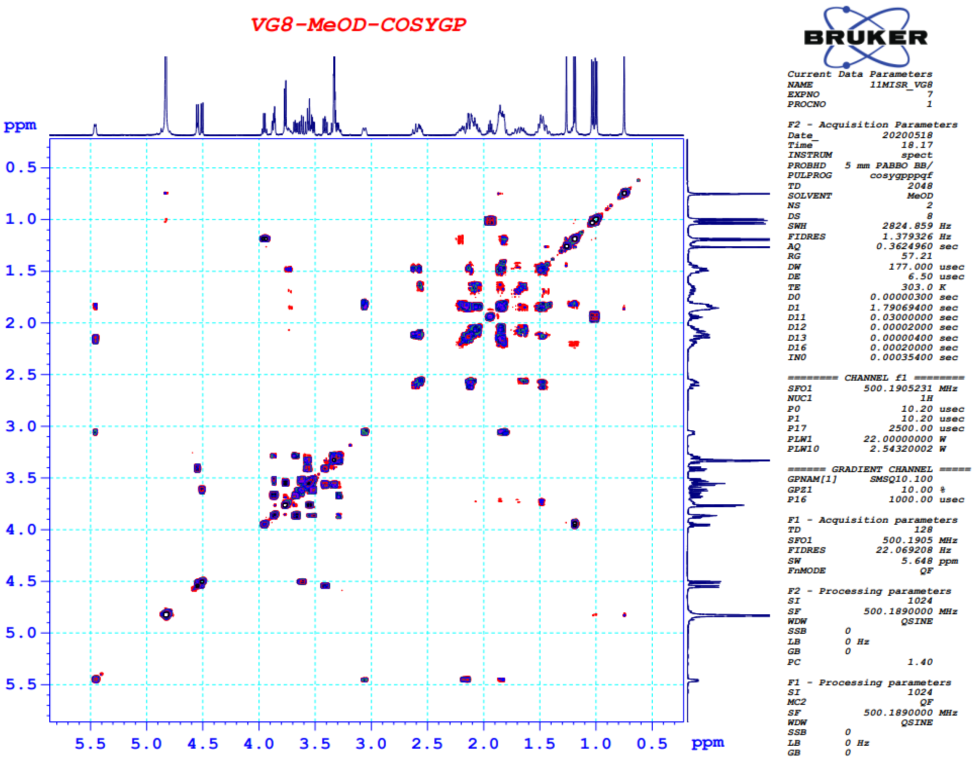


**Figure S17.** COSY spectrum of compound **3** (500/500 MHz, MeOD)

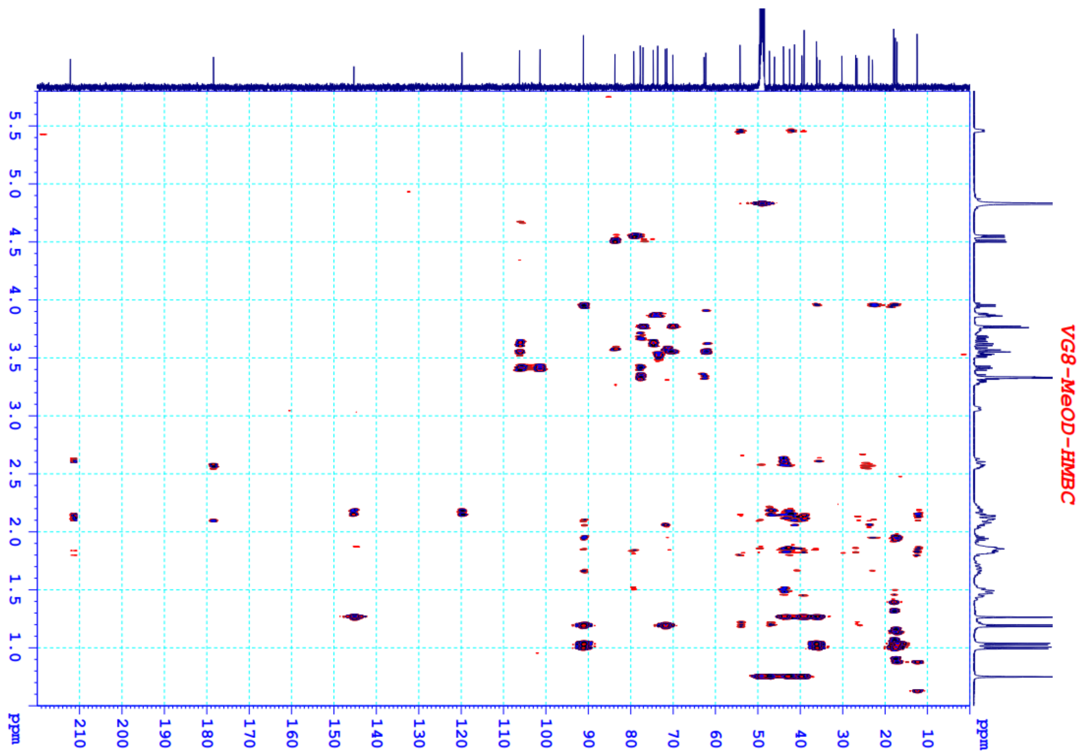


**Figure S 18.** HMBC spectrum of compound **3** (500/125 MHz, MeOD)

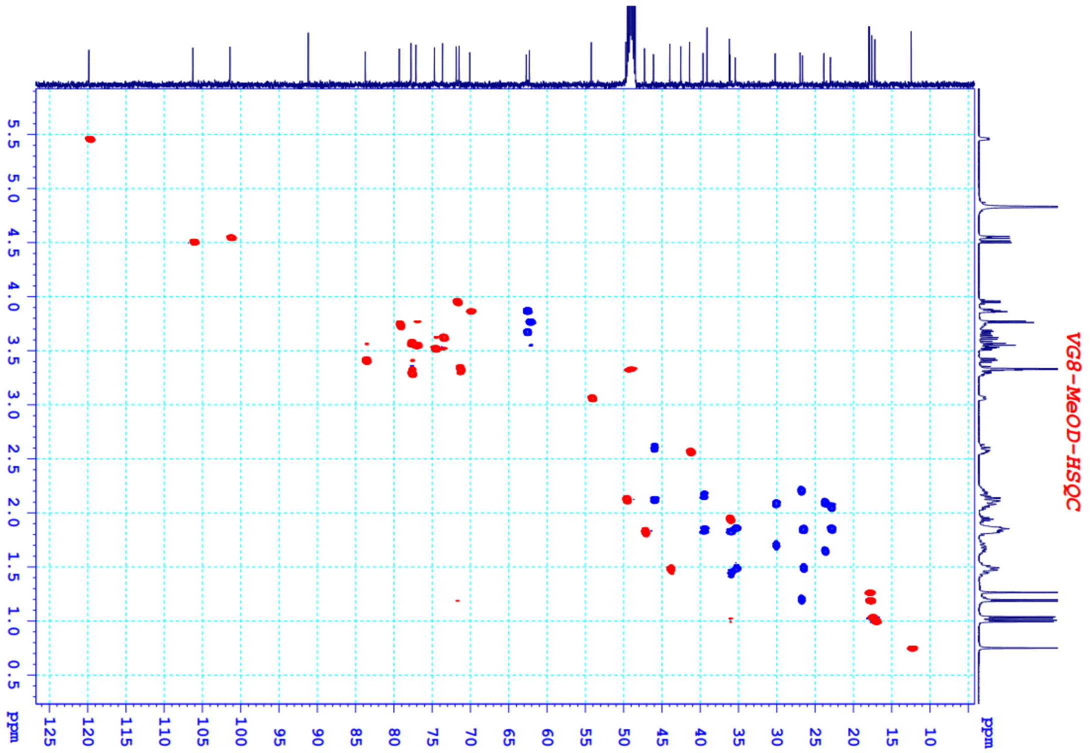


**Figure S19.** HSQC spectrum of compound **3** (500/125 MHz, MeOD)

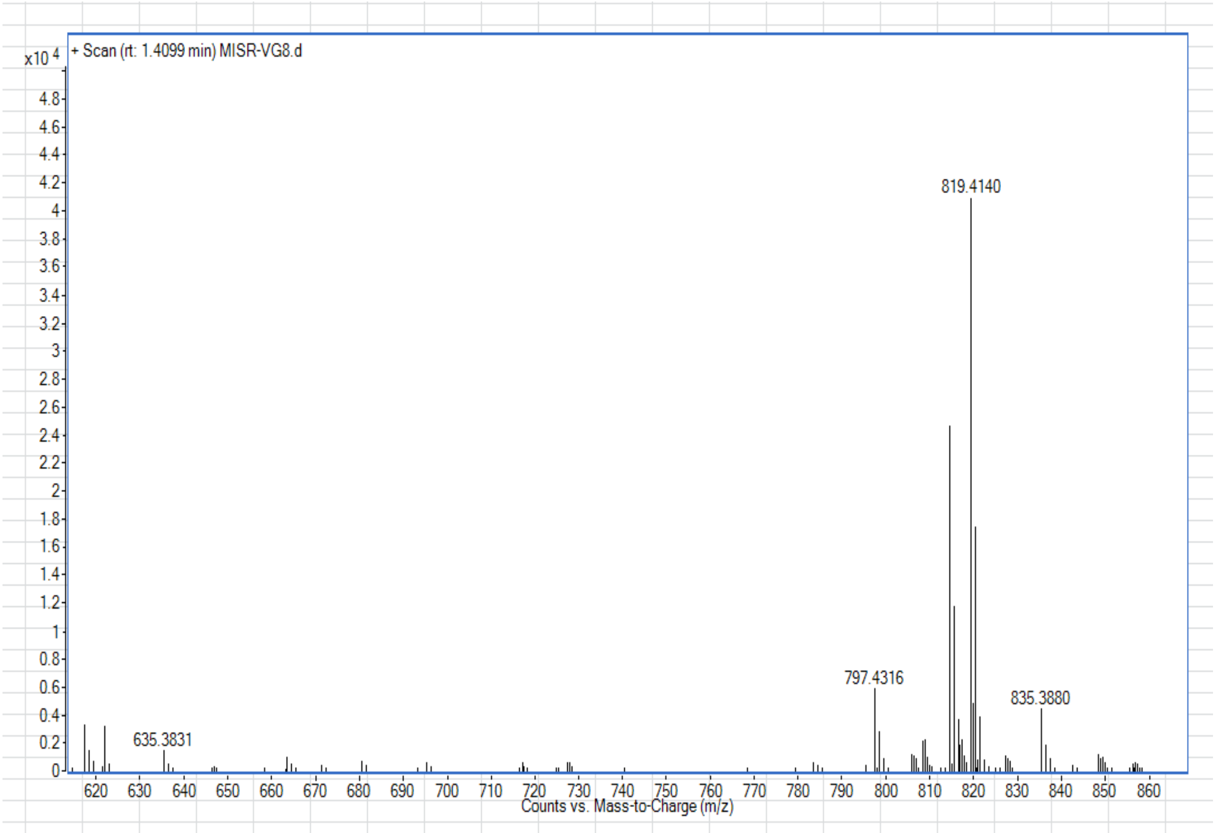


**Figure S 20.** HR-ESI-TOF-MS spectrum of compound **3**

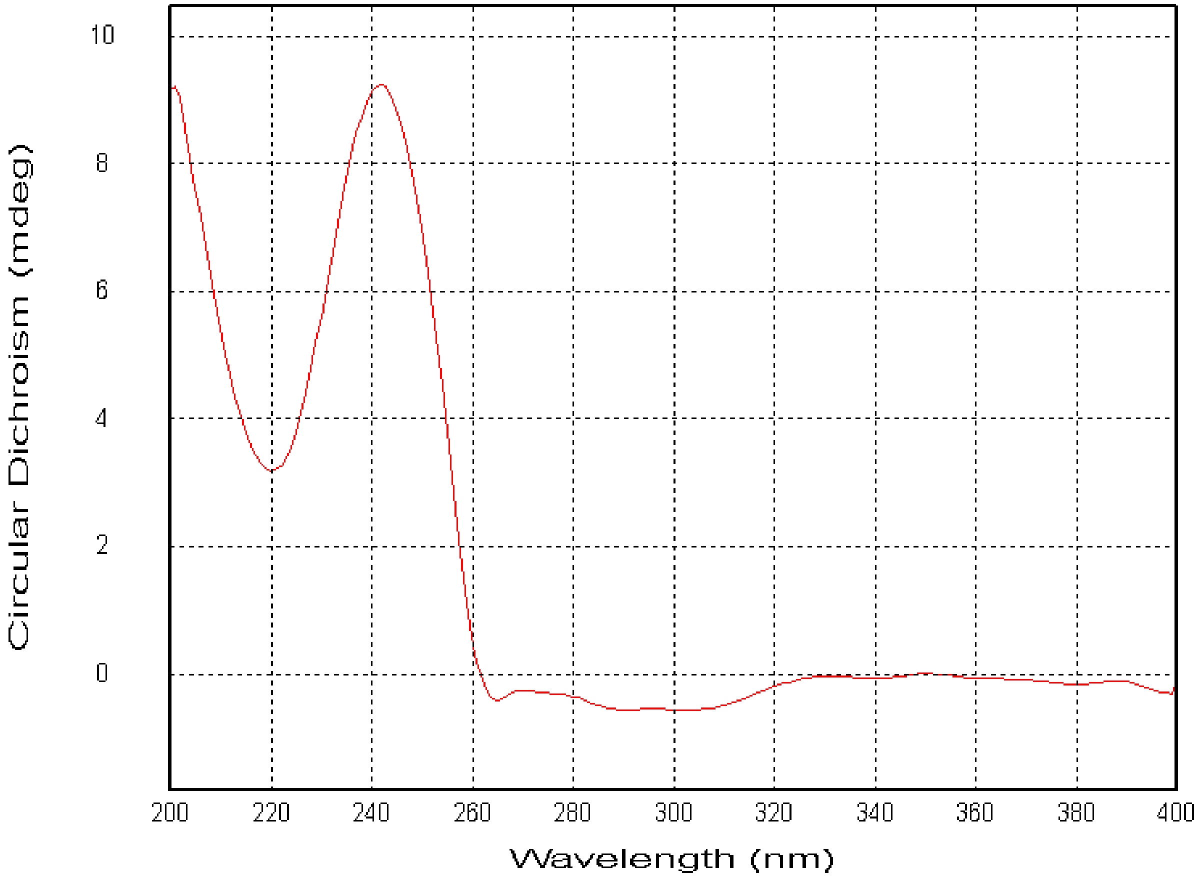


**Figure S 21.** CD spectrum of compound **3**
